# Dislocation of hemiarthroplasty after hip fracture is common and the risk is increased with posterior approach: result from a national cohort of 25,678 individuals in the Swedish Hip Arthroplasty Register

**DOI:** 10.1080/17453674.2021.1906517

**Published:** 2021-04-06

**Authors:** Ammar Jobory, Johan Kärrholm, Susanne Hansson, Kristina Åkesson, Cecilia Rogmark

**Affiliations:** a Department of Orthopaedics, Lund University, Skåne University Hospital, Malmö; b Swedish Hip Arthroplasty Register, Registercentrum Västra Götaland, Gothenburg; c Department of Orthopaedics, Institute of Clinical Sciences, Sahlgrenska Academy, University of Gothenburg, Sweden

## Abstract

Background and purpose — Reported revision rates due to dislocation after hemiarthroplasty span a wide range. Dislocations treated with closed reduction are rarely reported despite the fact that they can be expected to constitute most of the dislocations that occur. We aimed to describe the total dislocation rate on the national level, and to identify risk factors for dislocation.

Patients and methods — We co-processed a national cohort of 25,678 patients in the Swedish Hip Arthroplasty Register, with the National Patient Register (NPR) and Statistics Sweden. Dislocation was defined as the occurrence of any ICD-10 or procedural code related to hip dislocation recorded in the NPR, with a minimum of 1-year-follow-up. In theory, all early dislocations should thereby be traced, including those treated with closed reduction only.

Results — 366/13,769 (2.7%) patients operated on with direct lateral approach dislocated, compared with 850/11,834 (7.2%) of those with posterior approach. Posterior approach was the strongest risk factor for dislocation (OR = 2.7; 95% CI 2.3–3.1), followed by dementia (OR = 1.3; CI 1.1–1.5). The older the patients, the lower the risk of dislocation (OR = 0.98 per year of age; CI 0.98–1.0). Neither bipolar design nor cementless stems influenced the risk.

Interpretation — The choice of posterior approach and dementia was associated with an increased dislocation risk. When hips treated with closed reduction were identified, the frequency of dislocation with use of direct lateral and posterior approach more than doubled and tripled, respectively, compared with when only revisions due to dislocation are measured.

Displaced femoral neck fractures in elderly patients have traditionally been treated with hemiarthroplasty (HA). Dislocation of the prosthesis is a major complication, affecting 1.5–15% of patients (Enocson et al. 2008, Figved et al. 2009, Leonardsson et al. 2012, Bensen et al. 2014, Parker 2015, Svenoy et al. 2017). The varying rate may be explained by different surgical approach, follow-up time, age, and frailty of the patients. In addition, dislocation may be defined and reported in various ways, for example closed reduction, revision surgery, or both. A systematic review of 7 randomized trials, with a mix of approaches and 1–5 years’ follow-up time, reported a risk of revision due to dislocation of 3% (Burgers et al. 2012). Only open surgery due to dislocation (i.e., open reduction or revision) is reported in the Swedish Hip Arthroplasty (SHAR). By including closed reduction with a linkage to the National Patient Register (NPR) the under-reporting of dislocation can be highlighted.

Risk factors for dislocation can be divided into surgically related, implant-related, and patient-related factors. Posterior approach is a known surgically related risk factor (Varley and Parker 2004, Enocson et al. 2008, Leonardsson et al. 2012, Abram and Murray 2015, Svenoy et al. 2017). The risk is even higher if complete posterior repair is not performed (Enocson et al. 2008, Kim et al. 2016, Svenoy et al. 2017). Others are discrepancy of offset (Madanat et al. 2012, Mukka et al. 2015, Li et al. 2016) and, for elective THA, faulty positioning of the stem (McCollum and Gray 1990). Gjertsen et al. (2012) showed increased risk of revision because of dislocation if an uncemented technique was used compared with cementation, while other studies concluded no such association (Varley and Parker 2004, Figved et al. 2009, Abram and Murray 2015). The influence of the prosthetic design, uni- or bipolar head, on the risk of reoperation or dislocation in hip fracture patients is unclear. Several studies found no difference (Varley and Parker 2004, Enocson et al. 2008, 2012), while Leonardsson et al. (2012) showed increased risk of reoperation caused by dislocation with bipolar HA. For fracture patients, 2 studies (Li et al. 2016, Kristoffersen et al. 2020) reported dementia to increase the risk of dislocation while others (Ninh et al. 2009, Madanat et al. 2012, Abram and Murray 2015, Svenoy et al. 2017) did not. Neurological disease (Li et al. 2016) and dysplasia (Madanat et al. 2012, Mukka et al. 2015) are reported patient-related risk factors, whereas age, sex, and comorbidity do not seem to be associated with risk of dislocation (Enocson et al. 2008, 2012, Madanat et al. 2012, Abram and Murray 2015, Kim et al. 2016, Mukka et al. 2015, Svenoy et al. 2017). The influence of other possible confounders such as socioeconomic factors on the risk of dislocation have not, to our knowledge, been studied earlier.

We aimed to describe the total dislocation rate on a national level and to explore risk factors with possible influence on the dislocation rate.

## Patients and methods

SHAR is a national quality register for hip replacement operations in Sweden. SHAR has a coverage of 100% for all hospitals performing joint replacement surgery in Sweden, both public and private. Since 2005 hemiarthroplasties have also been reported with a completeness of approximately 96%. The completeness for reporting revisions (both HA and THA) is approximately 93% (Kärrholm et al. 2019). Open, but not closed, reductions of dislocations are reported to SHAR.

In the Swedish NPR, the Swedish National Board of Health and Welfare has collected data on diseases, surgical treatments, and medical care measures since 2001 (Ludvigsson et al. 2011). This includes all inpatients, both public and private hospitals, outpatient visits including day surgery, and psychiatric care from both private and public caregivers. Primary care is not yet covered in the NPR. Statistics Sweden (SCB) (2018) is responsible for official statistics in Sweden. SCB develops, produces, and disseminates statistics on Swedish residents and provides socioeconomic data, factors that can interact with both treatment decisions and outcome.

As identified in SHAR, hemiarthroplasty was used to treat 25,678 patients with acute hip fracture during 2005–2011 and these were included in this observational cohort study. Aided by the unique personal identity number given to each Swedish citizen, either at birth or on immigration, individuals can be cross-matched to NPR and SCB. ICD-10 codes were used for main and secondary diagnosis (WHO 2016). For procedural codes, NOMESCO codes (NOMESCO 2011) were used. The codes used to define hip prosthesis dislocation were M24.3-4, M24.4F, S73.0, T93.3, and all NOMESCO codes related to hip arthroplasty dislocation. Information concerning education and marital status was extracted from SCB.

A data set was created from the registers including patients with hip fracture treated with HA during 2005–2012. Since the majority of the dislocations occur within the 1st months after hemiarthroplasty surgery (Madanat et al. 2012, Gill et al. 2018), we decided on a minimum of 1-year follow-up, i.e., operations during 2012 were excluded. Only the 1st hip fracture surgery was included in patients with a 2nd contralateral hip surgery.

### Statistics

Possible risk factors and revision caused by dislocation were calculated using a chi-square test. The Elixhauser index was regrouped into 4 categories (0, 1, 2, and 3+) before analysis. Logistic regression analysis was used to evaluate the mutually adjusted effect of possible risk factors. This method was chosen because, due to the complexity of the final co-processed dataset, we had information only on whether a dislocation had occurred, not the date for such an event. The risk factors included in the logistic regression analysis were age, sex, surgical approach, cementation, prosthesis design (uni-/bipolar), dementia, Elixhauser in four categories, education, and civil status. Dementia was classified in SHAR as “none,” “suspect,” and “clear” cognitive impairment, based on a judgment of the patient and previous journals. In our analysis, “suspect” and “clear” were grouped together. 4,044 patients were excluded from this analysis because of missing data, mainly on dementia (Figure 1). 95% confidence interval is abbreviated as CI in the Results section.

**Figure:**
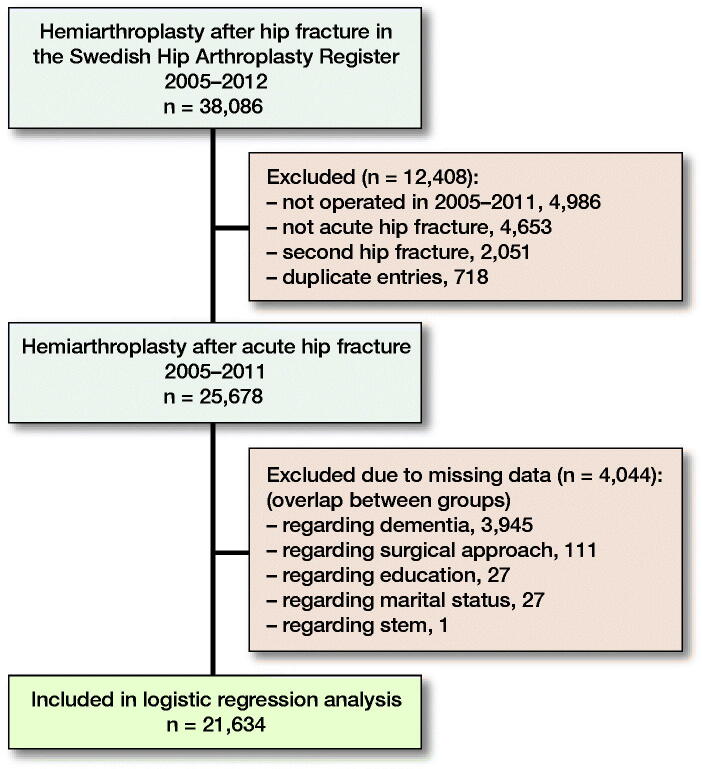
Flowchart for the study.

We used IBM SPSS Statistics 24 as statistical software (IBM Corp, Armonk, NY, USA).

### Ethics, funding, and potential conflicts of interest

The study was approved by the regional Ethical Review Board in Gothenburg, Sweden (271-14). The study adhered to the STROBE (Strengthening the Reporting of Observational Studies in Epidemiology) guidelines (von Elm et al. 2007). Due to Swedish legislation, data on individual study objects cannot be shared. This work was supported by grants from the Southern Health Care Region, and Swedish Research Council funding for clinical research in medicine, Sweden. No competing interests were declared.

## Results

Overall, the rate of dislocation was 1,220/25,678 (4.8%). Patients treated with posterior approach had a dislocation rate of 850/11,834 (7.2%) compared with 366/13,769 (2.7%) with direct lateral approach (Table 1).

**Table 1.** Patient characteristics and dislocation rate. Values are count (%)

	Dislocation		
	No	Yes	
	n = 24,458 (95)	n = 1,220 (5)	p-value
Age			< 0.001
< 75	2,101 (94)	135 (6)	
75–85	11,577 (95)	605 (5)	
> 85	10,780 (96)	480 (4)	
Sex			0.09
Female	17,347 (95)	838 (5)	
Male	7,111 (95)	382 (5)	
Surgical approach (missing 111)			< 0.001
Posterior	10,984 (93)	850 (7)	
Direct lateral	13,403 (97)	366 (3)	
Stem (missing 1)			0.7
Cemented	23,718 (95)	1,181 (5)	
Uncemented	739 (95)	39 (5)	
Head			< 0.001
Unipolar	12,463 (96)	529 (4)	
Bipolar	11,995 (95	691 (5)	
Dementia (missing 3,945)			0.005
No	14,357 (96)	647 (4)	
Yes	6,380 (95	349 (5)	
Elixhauser			0.08
0	15,345 (95)	733 (5)	
1	4,009 (95)	232 (5)	
2	2,534 (95)	133 (5)	
3	2,570 (96)	122 (4)	
Education (missing 27)			0.3
Primary school	14,777 (95)	766 (5)	
High school	6,519 (95)	311 (5)	
University	3,135 (96)	143 (4)	
Civil status (missing 27)			0.7
Partner	9,312 (95)	471 (5)	
Alone	15,119 (95)	749 (5)	

The overall frequency of HA revisions caused by dislocation as reported to SHAR was 1.6% (403/25,678). Patients treated with posterior approach had a frequency of 2.0% (241/11,834) and those treated with direct lateral approach a frequency of 1.2% (162/13,769; p < 0.001). Thus, the relative share of dislocations increased from 2.0% to 7.2% with use of a posterior incision and from 1.2% to 2.7% with use of a lateral approach when hips treated with closed reduction only were included.

Using logistic regression analysis, posterior approach was found to be the most pronounced risk factor for dislocation (OR = 2.7; CI 2.3–3.1) (Table 2). Higher age was associated with a lower risk of dislocation (OR = 0.98 per year of age; CI 0.98–1.0). In the 21,733 patients with complete data on cognitive status, dementia was associated with increased risk (OR = 1.3; CI 1.1–1.5).

**Table 2.** Logistic regression model (n = 21,634)

Factor	OR (95% CI)
Age (per year)	0.98 (0.98–1.0)
Male sex	1.1 (0.97–1.3)
Posterior approach	2.7 (2.3–3.1)
Cemented stem fixation	0.83 (0.58–1.2
Unipolar head	0.93 (0.82–1.1)
Dementia	1.3 (1.1–1.5)
Elixhauser (ref. 0)	
1	1.2 (0.97–1.4)
2	1.1 (0.86–1.3)
3	1.0 (0.82–1.3)
Education (ref. primary school)	
high school	0.93 (0.80–1.1)
university	1.0 (0.81–1.2)
Civil status (partner)	0.92 (0.80–1.1)

In a subgroup analysis of 6,709 patients with suspected or manifest cognitive impairment, 129/3,962 (3.3%) patients with direct lateral approach dislocated, compared with 220/2,747 (8.0%) of those with posterior approach.

Sex, education, or marital status choice of uni- or bipolar design and type of fixation had no statistically significant influence on the risk of dislocation. Subgroup analysis of prosthetic design showed that 32% of the unipolar (3,917/12,423) and 59% of the bipolar (7,031/11,928) hemiarthroplasties were operated with posterior approach. In most of the patients the stem had been fixed with bone cement (24,899/25,677 patients, 97%).

## Discussion

With a dislocation rate of 7% after posterior approach and 3% after direct lateral, our findings are on a par with earlier observations of increased risk dislocation with posterior approach compared with direct lateral approach (Enocson et al. 2008, Madanat et al. 2012, Abram and Murray 2015, Svenoy et al. 2017), but with the strength of a much larger cohort on a national level. Both our analysis and earlier studies showed the posterior approach to be the most important risk factor for dislocation (Varley and Parker 2004, Enocson et al. 2008, Abram and Murray 2015, Svenoy et al. 2017, Gill et al. 2018). Use of tendon-to-bone repair may reduce the risk of dislocation after a posterior approach (Enocson et al. 2008, Kim et al. 2016). Such a posterior repair has been practiced for many years in Sweden (Enocson et al. 2008), but its use is not reported to the SHAR. Most probably the risk of dislocation with use of the posterior approach as observed by us can be improved with use of posterior repair performed by experienced hip surgeons. These optimum conditions are, however, usually not present when hemiarthroplasty is performed as everyday practice in Sweden. Both residents and consultants do emergency hip fracture surgery. According to the Swedish Fracture Register (2018), approximately 25% of hemiarthroplasties are performed by residents, 20% by highly specialized trauma consultants, less than 5% by highly specialized arthroplasty consultants, and the rest by other consultants.

The direct lateral approach may be associated with other potential shortcomings such as lateral pain and a positive Trendelenburg test. To what extent this is clinically relevant for individuals with hip fracture or should be weighed against the increased dislocation risk observed after use of a posterior approach remains uncertain (Palan et al. 2009, Leonardsson et al. 2013, Kristensen et al. 2017, Mukka et al. 2017).

We found an overall dislocation rate of 5%. The SHAR reports only open treatment of a dislocation, i.e., not closed reduction in the emergency room or operating theatre. We found the revision rate caused by dislocation to be 1.6%. The outcome measure “revision due to dislocation” clearly underestimates the clinical problem with dislocation, as only 1 in 3 individuals with dislocation(s) had revision surgery. That 2/3 of patients with a dislocating hemiarthroplasty suffer recurrent dislocations (Madanat et al. 2012, Gill et al. 2018) underlines the problem with revision as outcome for fracture patients. Dislocation is not an absolute indication for revision surgery and exchange of implant parts. The risk of open surgery is weighed against the risk of forthcoming dislocations and the discomfort of the patients. Patients may be advised against secondary open surgery, or decline the proposal themselves. Nevertheless, repeated dislocations treated with closed reduction are usually devastating for the patient as reflected in reduced health-related quality of life (Enocson et al. 2009) and also result in additional hospital costs (Sanchez-Sotelo et al. 2006).

We found dementia to be associated with an increased risk of dislocation. This is in line with Li et al. (2016), but in contrast to other studies (Ninh et al. 2009, Madanat et al. 2012, Abram and Murray 2015, Kim et al. 2016, Svenoy et al. 2017). All these studies comprise smaller patient groups than observed by us and may lack statistical power. Individuals with either manifest dementia or suspicion of cognitive impairment showed increased risk of dislocation with the posterior approach compared with the direct lateral approach. Although commonly recommended, movement precautions and mandatory use of ADL equipment during the recovery phase do not affect the risk of dislocation when patients are operated on with the direct lateral approach (Jobory et al. 2019). Posterior approach has not, to our knowledge, been studied in this context, but many surgeons prescribe movement precautions and mandatory use of ADL equipment to avoid dislocation. Since patients with dementia will have difficulties following such precautions, this is another reason why the posterior approach should be particularly avoided in individuals with concomitant cognitive impairment.

Several studies have shown age not to be a risk factor (Ninh et al. 2009, Madanat et al. 2012, Abram and Murray 2015, Mukka et al. 2015, Svenoy et al. 2017). In contrast, we found that older patients have lower risk of dislocation. Another Swedish register study found that patients under 75 years of age were at higher risk of reoperation due to dislocations than those over 85 (Leonardsson et al. 2012). Younger patients may have a more active lifestyle, and are therefore more prone to dislocation. Using open secondary surgery as outcome measure can also introduce selection bias, as younger patients more often may be recommended reoperation, whilst old and frail patients more frequently may be treated with repeated closed reductions only. Therefore, we believe that our result, including virtually all dislocations, confirms the lower risk among the oldest.

We found no difference in dislocation risk between bipolar and unipolar HA in the logistic regression analysis. The difference in crude dislocation rate may be explained by the posterior approach being more common for bipolar HA, than for unipolar HA. Our observation is supported by earlier studies (Varley and Parker 2004, Enocson et al. 2008, 2012), but Leonardsson et al. (2012) found the bipolar design to be associated with increased risk of revision caused by dislocation. In agreement with earlier studies (Varley and Parker 2004, Figved et al. 2009, Abram and Murray 2015) we found no difference between cemented versus uncemented HA regarding the risk of dislocation.

Comorbidity seems not to affect the risk of dislocation, in accordance with previous studies (Madanat et al. 2012, Mukka et al. 2015, Kim et al. 2016, Svenoy et al. 2017). A smaller retrospective study reported male sex as a risk factor (Ninh et al. 2009), but neither we, nor several other studies (Enocson et al. 2008, 012, Leonardsson et al. 2012, Madanat et al. 2012, Abram and Murray 2015, Kim et al. 2016, Svenoy et al. 2017), identified sex as a risk factor.

Patient compliance usually influences the choice between THA and HA in clinical everyday life, partly because of the risk of dislocation. Lifestyle factors, including substance or alcohol abuse, can affect patient compliance. However, 2 studies found alcohol abuse disease not to affect the risk of dislocation (Madanat et al. 2012, Svenoy et al. 2017). Obtaining reliable data on whether a patient is addicted is difficult, as both medical records and register data will underestimate the problem. As blunt proxies for socioeconomic distress, we found that education level and marital status did not affect the dislocation rate.

The strength of our study is that it includes a large amount of material with a variety of patients and surgeons. We therefore believe that our data reflects everyday practice in Sweden, with good generalizability to the majority of other public healthcare systems. With the use of both diagnostic and therapeutic codes for hip dislocation and closed reduction we have tried to cover as many treatment occasions of hip dislocation as possible.

Nonetheless, our study also has limitations. We lack information on use of posterior soft-tissue repair or not, implant positioning (no access to postoperative radiographs), and the skills of the surgeons involved. All these factors have been reported to influence the dislocation rate (Enocson et al. 2008, 2012, Madanat et al. 2012, Abram and Murray 2015, Mukka et al. 2015, Kim et al. 2016, Svenoy et al. 2017). Further, information on dementia was missing in 1/6 of the patients. We might also have overestimated the dislocation rate because of lack of information on laterality in the NPR. This means that a dislocation on the opposite side might have been included in those with bilateral arthroplasties. Nevertheless, these events could be expected to be equally distributed between the groups studied, and our numbers are on a par with smaller clinical studies (Enocson et al. 2008, Madanat et al. 2012, Abram and Murray 2015, Svenoy et al. 2017). Finally, we could not account for any differences in time to follow-up between groups of patients with different presumed risk factors e.g., between male and female patients. Previous studies have, however, shown that dislocation is an early complication mainly occurring within the first postoperative months with decreasing incidence up to 1 year (Ninh et al. 2009, Madanat et al. 2012, Gill et al. 2018). Therefore, we think that the main conclusions from this study would have been the same if time to dislocation had been considered in the analyses.

We have not found any previous study analyzing the total dislocation rate on a national level. Our findings of a high dislocation rate in Sweden are unsatisfying and suggests that the results can be improved. Such an improvement would include several measures, not least improved surgical technique, especially with use of the posterior approach. We think that it is important to share our results with the orthopedic community and discuss it in the light of both limitations and strengths. One should realize that these patients have neither the time, nor the opportunity, to seek help at another hospital or to find an expert arthroplasty surgeon. Only if we point out how high the dislocation rate is will surgeons realize that “business as usual” is not good enough. There is then fertile soil for introducing better surgical technique(s) and better tutoring.

In conclusion, posterior approach for hemiarthroplasty is associated with a higher risk of dislocation compared with direct lateral approach. Patients with dementia have an increased risk. Our data refutes bipolar head as a risk factor for dislocation. Neither did socioeconomic factors or comorbidity play a role. 2 in 3 dislocations were treated with closed reduction only. The outcome measure “revision due to dislocation” underestimates the clinical problem with dislocation.
